# From Stories to Science: Mapping Global Trends in Narrative Medicine Research (2004–2024)

**DOI:** 10.7759/cureus.89211

**Published:** 2025-08-01

**Authors:** Guangbin Chen, Chunyan Xu, Ke Wang, Zhilin Wang, Guangming Xu, Xuelei Ji

**Affiliations:** 1 Department of Hepatobiliary Surgery, The Second People's Hospital of Wuhu, Wuhu Hospital Affiliated to East China Normal University, Wuhu, CHN; 2 Department of Teaching and Research, The Second People's Hospital of Wuhu, Wuhu Hospital Affiliated to East China Normal University, Wuhu, CHN; 3 Graduate School, Wannan Medical College, Wuhu, CHN

**Keywords:** bibliometric analysis, hotspots, medical humanities, narrative medicine, research trends

## Abstract

Narrative medicine is defined as a medical approach that utilizes the power of stories, both patients' illness narratives and healthcare providers' reflective accounts, to promote healing, foster empathy, and enhance the therapeutic relationship through close attention to the language, metaphor, and meaning embedded in illness experiences. Despite its growing importance in contemporary healthcare, comprehensive bibliometric analyses of narrative medicine research trends remain limited. This study aims to systematically map global research patterns, identify key contributors, and analyze thematic evolution in narrative medicine literature over the past two decades.

We conducted a comprehensive bibliometric analysis using the Citexs platform to examine narrative medicine research published from 2004 to 2024. The PubMed database was systematically searched using Boolean search terms: "narrative medicine OR medical storytelling OR clinical narrative OR patient-centered narrative OR healthcare narrative OR personalized medicine narrative". Inclusion criteria encompassed English-language articles only. Publication trends, geographic distribution, institutional productivity, author contributions, and thematic analysis were evaluated using advanced bibliometric techniques and the BioBERT biomedical language representation model for disease entity analysis.

A total of 28,029 English-language articles were identified, demonstrating exponential growth with a peak output of 5,063 articles in 2024. The United States led global research productivity with 7,933 articles (28.3%), followed by the United Kingdom (4,704 articles, 16.78%) and Italy (2,743 articles, 9.79%). The University of Toronto emerged as the most productive institution (432 publications). Keyword analysis revealed "systematic review", "COVID-19", "treatment", and "artificial intelligence" as the most frequent terms, indicating the field's responsiveness to contemporary healthcare challenges and technological integration. Disease entity analysis identified "Neoplasms" (5,282 articles), "Death" (4,948 articles), "Pain" (4,345 articles), "Inflammation" (4,338 articles), and "Depressive Disorder" (3,933 articles) as the most commonly studied conditions.

This bibliometric analysis demonstrates narrative medicine's transformation from a niche concept to a mainstream healthcare approach with substantial academic recognition. The exponential publication growth reflects increasing institutional support and clinical integration of narrative approaches. Geographic concentration in developed healthcare systems suggests opportunities for global expansion, particularly in culturally diverse contexts. The emergence of artificial intelligence as a research hotspot indicates the field's adaptive capacity to incorporate technological advances while maintaining humanistic principles. The predominance of cancer, death, pain, and mental health conditions underscores narrative medicine's particular relevance in addressing complex psychosocial dimensions of patient care that traditional biomedical approaches cannot fully capture. These findings emphasize narrative medicine's critical role in humanizing modern medical practice and its essential contribution to patient-centered care delivery.

## Editorial

Introduction and background

Narrative medicine is defined as a medical approach that utilizes the power of stories, both patients' illness narratives and healthcare providers' reflective accounts, to promote healing, foster empathy, and enhance the therapeutic relationship through close attention to the language, metaphor, and meaning embedded in illness experiences. This discipline emphasizes the fundamental importance of patient stories in clinical practice and has garnered substantial attention in contemporary healthcare discourse [1,2]. This innovative approach represents a paradigmatic shift from the traditional biomedical model toward a more holistic understanding of illness and healing, recognizing that disease affects not merely biological systems but entire human beings embedded within complex social, cultural, and emotional contexts [3,4].

The conceptual foundations of narrative medicine can be traced to the pioneering work of Rita Charon at Columbia University, who first articulated the discipline's core principles in the early 2000s [5]. Charon's seminal contributions established narrative medicine as a rigorous academic field that combines close reading skills from literary criticism with clinical observation and empathetic engagement. This interdisciplinary approach fosters empathy, enhances patient-physician communication, and contributes to more personalized and compassionate care delivery [6,7].

The theoretical underpinnings of narrative medicine draw from phenomenology, hermeneutics, and narrative psychology, recognizing that human experience is fundamentally narrative in structure [8,9]. Patients do not simply present with symptoms; they arrive with stories - complex accounts of their illness experience that encompass physical manifestations, fears, hopes, cultural beliefs, and existential concerns [10]. By systematically valuing patients' lived experiences and personal narratives, narrative medicine effectively bridges the gap between objective clinical data and the humanistic dimensions of healthcare [11].

The growing recognition of narrative medicine's importance has been catalyzed by several factors within contemporary healthcare: increasing awareness of healthcare disparities requiring culturally responsive care, the perceived dehumanization of evidence-based medicine, and the emphasis on patient-centered care and shared decision-making [12,13]. Moreover, narrative medicine has demonstrated measurable impacts, including improved physician empathy, enhanced communication skills, reduced burnout among healthcare providers, and increased patient satisfaction [14,15]. To address the notable lack of comprehensive bibliometric reviews in this rapidly evolving field, we conducted a systematic bibliometric analysis of narrative medicine research published over the past two decades.

Methodology

The Citexs platform (https://www.citexs.com/) provides bibliometric analysis that can conduct literature analysis and big data mining based on the PubMed literature database, tracing the developmental history of a field, analyzing changes in research topic hotspots, and visualizing the analysis results. We performed a comprehensive search of the PubMed database employing the following Boolean search strategy: "narrative medicine OR medical storytelling OR clinical narrative OR patient-centered narrative OR healthcare narrative OR personalized medicine narrative". Our literature retrieval, completed on January 14, 2025, encompassed research publications spanning from January 2004 to December 2024. We applied stringent inclusion criteria, restricting our search to English-language publications and considering only document types classified as "articles". This comprehensive search strategy yielded a total of 28,029 publications for analysis.

Results and discussion

Publication Trends and Geographic Distribution

**Figure 1:**
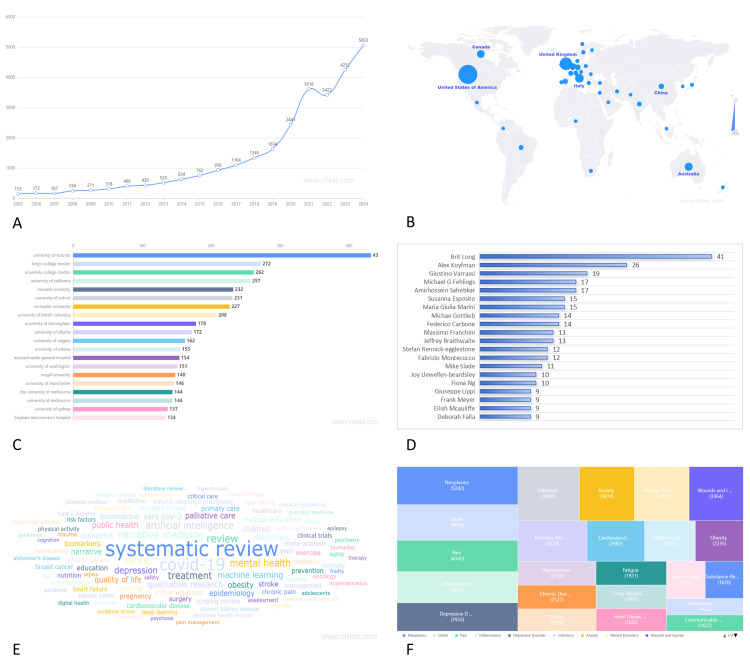
Comprehensive bibliometric analysis of narrative medicine research (2004-2024). (A) Annual publication trends demonstrating exponential growth in narrative medicine research. (B) Geographic distribution of publications by country, highlighting global research contributions. (C) Top-performing research institutions ranked by publication output. (D) Most prolific authors in narrative medicine research. (E) Frequency analysis of keywords revealing thematic evolution and research hotspots. (F) Disease entity analysis showing the most commonly studied medical conditions in narrative medicine research. Image credits: Guangbin Chen.

Our bibliometric analysis revealed several significant and noteworthy trends in narrative medicine research. Publication volume demonstrated consistent and steady growth over the study period, reaching its zenith in 2024 with 5,063 articles. Notably, the highest annual growth rate of 53.29% was observed in 2008 (Figure 1A), indicating a surge in scholarly interest and institutional recognition of narrative medicine's pivotal role in modern healthcare delivery.

Geographic analysis revealed that the United States dominated publication output with 7,933 articles (28.3% of total publications), followed by the United Kingdom (4,704 articles, 16.78%) and Italy (2,743 articles, 9.79%) (Figure 1B). This distribution reflects the strong emphasis on medical humanities and patient-centered care in these healthcare systems.

Institutional and Author Contributions

Analysis of institutional productivity revealed that the University of Toronto led global research output with 432 publications, followed by King's College London (272 publications), University College London (262 publications), University of California (257 publications), and Monash University (232 publications) (Figure 1C). These institutions represent centers of excellence in medical humanities and narrative medicine education.

The most prolific authors in the field were Brit Long (41 publications), Alex Koyfman (26 publications), Giustino Varrassi (19 publications), Michael G. Fehlings (17 publications), and Amirhossein Sahebkar (17 publications) (Figure 1D), demonstrating sustained scholarly commitment to advancing narrative medicine research.

Thematic Evolution and Research Hotspots

Keyword analysis serves as a powerful tool for identifying thematic evolution and research hotspots within a given field of study over time. Figure 1E illustrates the five most frequently occurring terms: "systematic review", "COVID-19", "review", "treatment", and "artificial intelligence". The prominence of "COVID-19" reflects the field's responsiveness to contemporary healthcare challenges, while "artificial intelligence" suggests emerging intersections between narrative medicine and digital health technologies.

Utilizing the BioBERT biomedical language representation model to analyze disease entities within the abstracts, we identified that "Neoplasms" (5,282 articles), "Death" (4,948 articles), "Pain" (4,345 articles), "Inflammation" (4,338 articles), and "Depressive Disorder" (3,933 articles) were the most commonly studied conditions (Figure 1F). This distribution highlights narrative medicine's particular relevance in addressing complex, chronic, and psychologically challenging medical conditions.

Implications and Future Directions

The findings from our bibliometric analysis underscore several critical implications for the field of narrative medicine and illuminate promising avenues for future research and implementation. The exponential growth in publications demonstrates the field's maturation and increasing recognition within academic medicine, suggesting a fundamental shift in how the medical community perceives the relationship between storytelling and healing [16,17]. This growth trajectory indicates that narrative medicine has successfully transcended its initial positioning as a niche subspecialty to become an integral component of contemporary healthcare discourse.

The geographic concentration of research, while highlighting centers of excellence in North America and Europe, simultaneously reveals significant opportunities for expanding narrative medicine initiatives globally, particularly in developing healthcare systems where cultural storytelling traditions may offer unique insights into healing practices [18,19]. Future research should prioritize cross-cultural investigations that explore how narrative medicine principles can be adapted across diverse healthcare contexts while respecting local cultural values.

The thematic evolution toward incorporating artificial intelligence and digital health technologies indicates the field's adaptive capacity in addressing emerging healthcare challenges. The integration of natural language processing and machine learning presents opportunities for scaling narrative medicine interventions while maintaining their therapeutic qualities [20,21]. Future research should explore how artificial intelligence can augment human narrative competence, enabling more sophisticated analysis of patient stories and personalized narrative interventions [22].

The predominance of cancer, death, pain, and mental health conditions in narrative medicine research reflects the field's particular strength in addressing existentially challenging medical situations where traditional biomedical approaches may prove insufficient [23,24]. Future investigations should expand this focus to include emerging health challenges such as long COVID, digital health equity, and the psychosocial consequences of medical artificial intelligence implementation.

Educational implications suggest an urgent need for systematic integration of narrative medicine curricula across medical training programs globally. Future research should prioritize the development of standardized narrative competency assessments and evidence-based pedagogical approaches that can be adapted across diverse educational contexts [25,26]. Additionally, interprofessional narrative medicine education programs should be developed to enhance collaborative care approaches and improve communication across healthcare teams [27].

Conclusion

Narrative medicine stands at the crucial intersection of clinical practice and humanistic inquiry, offering invaluable insights into patient experiences and healthcare delivery. Our comprehensive bibliometric study highlights the dynamic and rapidly expanding nature of this field, emphasizing its critical role in fostering empathetic, patient-centered, and effective healthcare. The field's evolution from a conceptual framework to an evidence-based discipline with measurable clinical impacts demonstrates its fundamental importance in addressing the humanistic dimensions of medical practice that traditional biomedical approaches cannot fully capture.

As healthcare systems worldwide grapple with challenges, including provider burnout, patient dissatisfaction, health inequities, and the integration of emerging technologies, narrative medicine offers essential tools for maintaining the human element in healing relationships. Continued research endeavors and interdisciplinary collaboration will be pivotal in harnessing the full transformative potential of narrative medicine to revolutionize patient care, medical education, and healthcare policy in the 21st century. The future of medicine lies not in choosing between scientific rigor and humanistic compassion, but in their thoughtful integration, a goal that narrative medicine continues to advance with increasing sophistication and empirical support.
